# Assessment of Typical Heavy Metals in Human Hair of Different Age Groups and Foodstuffs in Beijing, China

**DOI:** 10.3390/ijerph14080914

**Published:** 2017-08-14

**Authors:** Gang Liang, Ligang Pan, Xinhui Liu

**Affiliations:** 1 Beijing Research Center for Agricultural Standards and Testing, Beijing Academy of Agriculture and Forestry Science, Beijing 100097, China; liangg@brcast.org.cn; 2 Risk Assessment Lab for Agro-Products, Ministry of Agriculture, Beijing 100097, China; 3 State Key Laboratory of Water Environment Simulation, School of Environment, Beijing Normal University, Beijing 100875, China

**Keywords:** human hair, heavy metals, age groups, bio-indicator

## Abstract

Human hair of different age groups and foodstuff samples were collected in Beijing, China. The concerned metals—Cd, Cr, Pb, As, and Hg—were analyzed, and the metal levels in relation to age, gender, and dietary intake were further assessed. Results showed the highest level of the metals was shown by Pb, with an average concentration of 1.557 ± 0.779 mg/kg, followed by Cr (0.782 ± 0.394), Hg (0.284 ± 0.094), As (0.127 ± 0.078), and Cd (0.071 ± 0.032), following a decreasing order of Pb > Cr > Hg > As > Cd, which were all below the upper limit of normal values in China. The heavy metal concentrations varied greatly among different age groups, and higher concentrations for Cd, Cr, Pb, and As appeared in female hair, whereas higher Hg concentration were found in male hair, suggesting that age and gender were not crucial factors for assessing metal concentrations in human hair. The ingestion of cereals and vegetables were the main route by which heavy metals in the environment create hazardous health effects for local inhabitants, but the estimated metal intakes through food consumption were all lower than the proposed limit of Provisional Tolerable Weekly Intake (PTWI), indicating that heavy metals posed no health risks for the inhabitants. Furthermore, little relationship was found between metal intakes and the corresponding metal levels in hair. Nevertheless, the results of this study can be used to analyze the internal heavy metal burden in the resident population of Beijing area and can also serve as reference for further studies.

## 1. Introduction

Heavy metals are a class of non-biodegradable pollutants in the environment that can enter into human bodies through different routes, such as food consumption (which is also the main route of exposure to the heavy metals [1,2,3]), and then can be accumulated in the body [4]. Many of these metals are essential micronutrients (such as Fe, Cu, Zn, Cr, and As), but they can become toxic at concentrations higher than the amount normally required [5], whereas some non-essential micronutrients (such as Cd, Hg, and Pb), which have unknowing roles in living organisms, are toxic even at very low concentrations [6,7], eventually posing a serious health risk to human and ecosystem health [8,9]. For instance, chronic cadium intoxication may give rise to renal tubular dysfunction, anemia, and skeletal damage (itai-itai disease) [10,11]. Long-term exposure to lead may cause kidney and liver damage and has an adverse effect on the central and peripheral nervous systems, haemopoietic system, and cardiovascular system [12]. Therefore, it is important to monitor heavy metal levels, which are very important for assessing the potential health risks of metals to humans [13].

However, studies show that the analysis of air, water, and soil samples yield data that is worthless for assessing the health hazards of environmental pollution, as the actual degree of heavy metal contamination may vary widely in a given area [14]. Accordingly, the biological monitoring of heavy metals pollution for human exposure is of the optimal choice for researchers. For many years, hair analysis has been understood to play an important role in the monitoring of heavy metals [15,16] and has been considered one of the most important biomarkers according to the Environmental Protection Agency (EPA) [17]. As an excretory system, human hair can accumulate heavy metals [5,18], and as metabolic products, it can incorporate metals into its structure during its growth process [19]. Therefore, concentrations of heavy metals in hair can reflect the mean level in the human body, recording the population’s exposure to heavy metals [20,21]. Furthermore, compared to the other bio-indicators such as blood [22], nail [17], urine [23], and saliva [24], human hair has attractive advantages, such as finer stability, easy collection and transportation, convenient storage, and reflects long-time exposure to heavy metals and higher metal concentrations than in other bio-indicators [25]. Therefore, human hair is considered a good screening tool to extensively assess heavy metals levels [26,27,28]. So far, a number of recent studies reported the use of hair analysis to obtain information on heavy metal levels. For example, Ferré-Huguet et al. investigated the exposure to toxic metals of school children of 12–14 years living near a hazardous waste incinerator in Catalonia [26]. Wang et al. compared the trace elements and heavy metals concentrations of hair samples collected from an electronic waste (e-waste) recycling area and control areas [27]. Caroli et al. analyzed 19 minor and trace elements in hair of subjects aged 3–15 from several urban areas in Rome for assessing the reference values [29].

Beijing has experienced rapid economic development in the process of urbanization and industrialization during the past decades, inevitably producing heavy metal pollution issues, which have considerable influence on human health. However, there is no research data reported on the biological monitoring of heavy metal levels in human hair, which can directly reflect the status of heavy metals in the human body. Thus, the main objectives of our study were (1) to determine the concerned toxic metal concentrations in human hair and foodstuff samples, (2) to assess the potential health risks of heavy metal exposure to the local inhabitants, and (3) to investigate if possible correlations between metal concentrations and age, genders, and metal dietary intakes exist.

## 2. Experiment

### 2.1. Sample Collection

In the present study, 225 human hair samples were collected from the healthy inhabitants of Beijing. All the participants consented to the test and were divided into five age groups: 7–12 years (I) (Male: 23, Female: 22 ), 13–18 years (II) (Male: 23, Female: 22), 19–35 years (III) (Male: 22, Female: 22), 36–50 years (IV) (Male: 25, Female: 23), and 51–75 years (V) (Male: 22, Female: 21). Each individual also filled out a questionnaire requesting information about the following variables: age, gender, and most consumed foodstuffs. Hair samples were obtained from people who did not have colored or treated hair. Because males had short hair, the hair was cut from all areas of the scalp. For females with long hair, only the distal portion of hair was cut. The hair samples were stored in polyethylene bags at room temperature until analysis. In parallel with the collection of hair samples, the most frequently consumed foodstuffs were also collected, such as fish (carp, white amur, crucian, hairtail, shrimp), vegetables (potato, cabbage, haricot bean, eggplant, white gourd, cucumber, cauliflower, carrot, onion), cereals (flour, rice, millet, corn), meat (pork, beef, mutton, chicken), and fruits (apple, pear, banana).

### 2.2. Chemicals and Materials

Measurements for total concentrations of Cd, Cr, and Pb were made on Inductively Coupled Plasma-Atomic Emission Spectrometry instrument (ICP-AES) with axial viewing configuration. Atomic Fluorescence Spectrometry (AFS-930) (Beijing Jitian Instrument Ltd., Beijing, China) was performed to obtain total concentrations of As and Hg. All reagents and standards were of ultrapure reagent grade unless otherwise specified, and all solutions were prepared in Millipore Milli-Q water. All glassware and plastic ware was pre-washed three times with deionized water and then soaked in 30% (v/v) HNO_3_ overnight. After soaking, the glassware and plastic ware were rinsed three times with deionized water again and dried in an oven. The certifacated reference material (CRM) human hair (GBW 07601) was obtained from the Shanghai Institute of Nuclear Research, Shanghai, China.

### 2.3. Sample Pre-Treatment

Consolidated procedures were utilized to pretreat the collected samples, including gentle removal of exogenous contaminants [30,31,32,33]. Hair samples were cut into pieces as small as possible and washed three times under continuous stirring with a mixture of ethyl ether/acetone (3:1 v/v), followed by soaking in a 5% EDTA solution for 30 min. Samples were then rinsed three times with de-ionized water, dried in an oven at 80 °C to constant weight, and stored in polyethylene bags. Vegetables and fruits were washed three times with de-ionized water before being cut into slices and then dried in an oven at 80 °C to constant weight. The dried samples were ground in a porcelain mortar, passed through a 200 mesh sieve, and stored in polyethylene bags. Fresh samples of fish were frozen at −20 °C until dissection. The muscle samples of each fish were removed with a plastic knife, homogenized and weighed, and then individual samples were dried to constant weight at 80 °C in acid-washed Petri dishes. A similar method was used to pretreat the samples of meat. The cereals samples were washed with de-ionized water and dried in an oven at 80 °C to constant weight prior to use.

### 2.4. Analytical Methods

A 0.5 g portion of the prepared sample was accurately weighed by using a four-decimal-place analytical balance and transferred to a screw-cap Polytetrafluoroethylene digestion vessel. A mixture of 2 mL nitric acid and 1 mL of 30% hydrogen peroxide was added at room temperature and left to predigest for 12 h. The vessels were then sealed and placed in the oven of 160 °C for 4 h. Once the digestion was complete, the vessels were allowed to cool to room temperature. The digest was diluted to 10 mL with Milli-Q water and ready for ICP-AES analysis. For analysis of As and Hg, 1 mL of the diluted solution was added to a 5 mL vessel, then 1 mL 5% thiocarbamide and 3 mL 10% nitric acid were added and left to react for 5 h at room temperature.

### 2.5. Quality Assurance and Control

The reagent blank samples were digested in the same way as for hair samples and were used to correct the instrument readings. The method limits of detection (MLOD) was estimated based on three times the standard deviation for digestion blanks (*n* = 5). The MLODs for Cd, Cr, and Pb were 0.01, 0.05, and 0.12 mg/kg, respectively, and for As and Hg were 0.40 and 0.25 μg/kg, respectively. The accuracy of the methods was validated by five replicate measurements. The standards reference materials GBW 07601 No. 5 was used to ensure the measurement precision and accuracy in the whole analytical procedure. The results of the observed metal concentration were in excellent agreement with the certified values and the satisfactory recoveries were 94 ± 8%, 102 ± 11%, 99 ± 8%, 103 ± 10%, and 103 ± 9% for Cd, Cr, Pb, As, and Hg, respectively.

### 2.6. Statistical Analysis

Statistical analysis data treatment and analysis were performed with SPSS 13.0 package (SPSS Inc., Chicago, IL, USA). Gender difference in concentration of the selected heavy metals in scalp hair was tested by Student’s *t* test. The Pearson’s correlation coefficient was calculated to investigate relationship between the heavy metal concentrations recorded in hair and age. Linear regression analysis was also performed to quantify relationships between levels of heavy metals in hair and age. To test the difference of heavy metal concentrations in hair between different age groups, one-way ANOVA (Analysis of Variance) was used. A *p* value of less than 0.05 was assumed to be statistically significant.

## 3. Results and Discussion

### 3.1. Average Concentrations of Heavy Metals in Hair Samples

The average concentrations of the selected heavy metals along with relevant standard deviation (SD) values are listed in Table 1. As shown in Table 1, there was a striking difference in the concentration of different heavy metals: the average concentrations for Cd, Cr, Pb, As, and Hg were 0.071, 0.782, 1.557, 0.127, and 0.284 mg/kg with an uncertainty of 0.032, 0.394, 0.779, 0.078, and 0.094 mg/kg, respectively. The average concentrations of the studied heavy metals in human hair were all below the upper limit of normal values in China as well [34]. Compared with the average values, the SD values are relatively large, but the SD values merely reflect the distribution of the heavy metal concentrations due to the biological variability of each individual. In addition, there was also a distinguishable change in the concentrations of the selected heavy metals in the hair samples, and the concentrations for Cd, Cr, Pb, As, and Hg varied from 0.02 to 0.96, 0.05 to 4.54, 0.11 to 6.65, 0.03 to 0.87, and 0.04 to 0.87 mg/kg, respectively, which were all within the reference concentration range reported previously [35]. For comparison, the hair metal concentrations of different countries are also listed in Table 2. According to Table 2, the Cr and As concentrations in hair of Beijing inhabitants were relatively higher than most of the countries listed; Pd and Cr concentrations were in the median range, and only Hg concentrations were comparative low to most other countries; therefore, the exposure levels of heavy metals to the inhabitants living in Beijing should be paid more attention to in the future.

For the studied heavy metals, Pb presented the highest concentration in hair, while Cd presented the lowest: Pb concentration was almost 22 times as much as that of Cd. The components in hair were arranged in the following decreased concentration order: Pb > Cr > Hg > As > Cd. This is in agreement with previous studies in China [34], but is not consistent with the previous reported results of other countries, as shown in Table 2. This could be mainly due to differences of geographical environment and dietary habits [25,39,40]. Furthermore, in order to investigate the correlation between the studied heavy metal concentrations recorded in the scalp hair, the Pearson’s correlation coefficient was calculated and is listed in Table 3. The results show that there is no significant correlation between the studied metals: a correlation was only found slightly between hair Pb and As concentration (*R*^2^ = 0.399, *p* < 0.01).

### 3.2. Heavy Metal Concentrations in Human Hair of Different Gender and Age Groups

**Figure 1:**
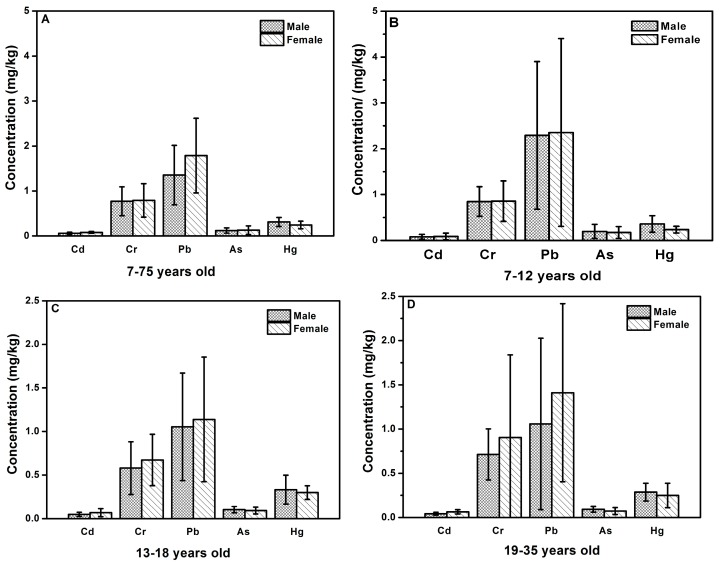

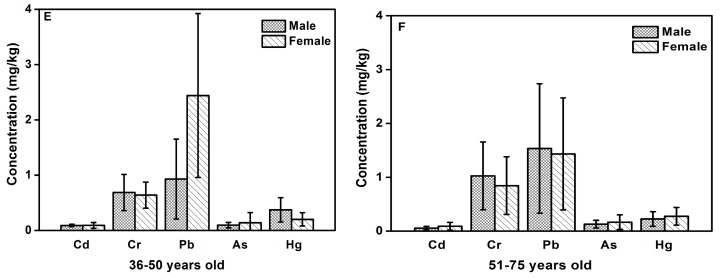
Comparison of hair metal concentrations between genders of different age groups. (**A**) 7–75 years old; (**B**) 7–12 years old; (**C**) 13–18 years old; (**D**) 19–35 years old; (**E**) 36–50 years old; (**F**) 51–75 years old.

Figure 1 shows an overall view of the main outcome of this study according to gender and age groups for the selected metals measured in the hair samples. The average heavy metal concentrations for males and femalse are presented in Figure 1A. As shown in Figure 1A, a striking difference exists in the different heavy metal levels. Pb presented in the highest concentration, and Cd presented in the lowest. Interestingly, the same decreased order of the selected heavy metals was also observed for both male and female: Pb > Cr > Hg > As > Cd. Moreover, it also showed the difference between hair metal concentrations in terms of gender. For the studied heavy metals, variations of concentrations according to gender were not appreciable, except Pb (1.355 mg/kg for male, 1.788 mg/kg for female), which was well in accordance with previous research [36]. Pearson’s correlation analysis also showed no significant difference between heavy metal levels in hair and gender (*p* > 0.05). Furthermore, women exhibited higher concentration of the studied metals (Pb, Cr, As, and Cd) than men exhibited, whereas mercury concentration in the hair of males was higher than that of females. These results also showed that gender could not to be considered as a critical factor to determine metals accumulation in human hair [41].

The average heavy metal concentrations of different age groups are presented in Figure 1B–F. Certainly, more information can be deduced by comparing subgroups of subjects based on age and gender. As shown in Figure 1, no distinct patterns of the metal concentration distribution were observed for males and females who were in the same age group. For example, the average levels of Cr and Pb in male hair samples was lower than that in female hair samples, whereas for Cd, the average levels for males were higher than that for females in all the five age groups. In addition, the highest concentrations for Cd, Cr, Pb, As, and Hg were in the 36–50, 51–75, 7–12, 7–12, and 35–50 age groups for males, and were in the 36–50, 19–35, 36–50, 7–12, and 13–18 age groups for females, respectively. These distribution differences in the heavy metals levels among the age groups were mainly due to the metabolic process, environmental exposure and physiological factors of gender [42,43]. Furthermore, there were little differences in Cd, Cr, and Hg concentrations between males and females who were in the same age group, and the results also confirmed that gender was not a crucial factor for assessing metal concentrations in human hair.

**Figure 2:**
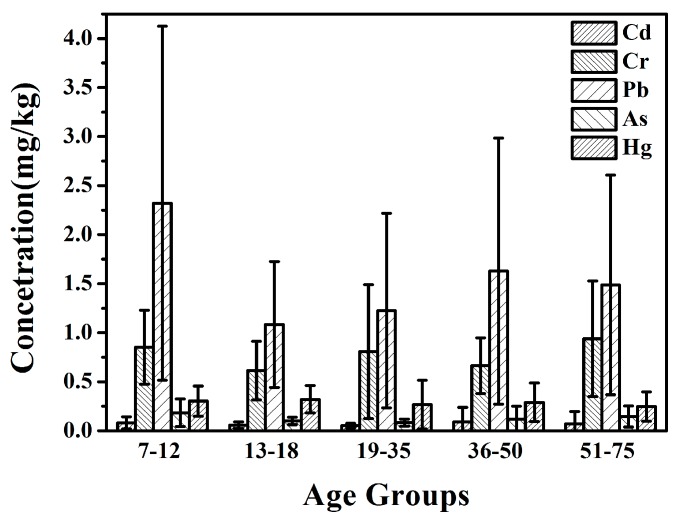
Concentrations of hair heavy metals of different age groups.

The mean metal concentration in different age groups (I–V) is shown in Figure 2. It showed that the metal concentrations varied as the age group with different tendency. The concentrations decreased in the order of IV > I > V > II > III for Cd, V > I > III > IV > II for Cr, I > IV > V > III > II for Pb, I > V > IV > II > III for As, II > I > IV > III > V for Hg, respectively. Therefore, individuals in the 13–18, 51–75, 7–12, 35–50, and 7–12 age groups were the ones with the highest concentrations of Hg, Cr, As, Cdm and Pb, respectively. According to previous studies, the variation of levels for different heavy metals from different age groups were related to the cumulative behavior of metals, life style, dietary habits, etc. [28,39]. ANOVA analysis showed that no significant differences in metal concentrations were found for heavy metals Cd and Hg (*p* > 0.05) between different age groups. However, the mean values of Pb were obviously greater than the other four heavy metals, and the concentrations for the studied metals decreased in the same order of Pb > Cr > Hg > As > Cd in all the five subgroups.

Furthermore, in agreement with other authors [36,44], the regression analysis did not show any relationship between the age and the metal concentrations in hair. Pearson’s correlation analysis showed that there was no significant correlation between the metal concentrations and the age groups (*p* > 0.05) except Pb for males (*p* < 0.05) and As for females (*p* < 0.05). In addition, no clear trend can be identified as a function of gender for the studied heavy metals as well.

### 3.3. Health Risks of Heavy Metal Intakes, and Relationship with Hair Metal Concentrations

**Figure 3:**
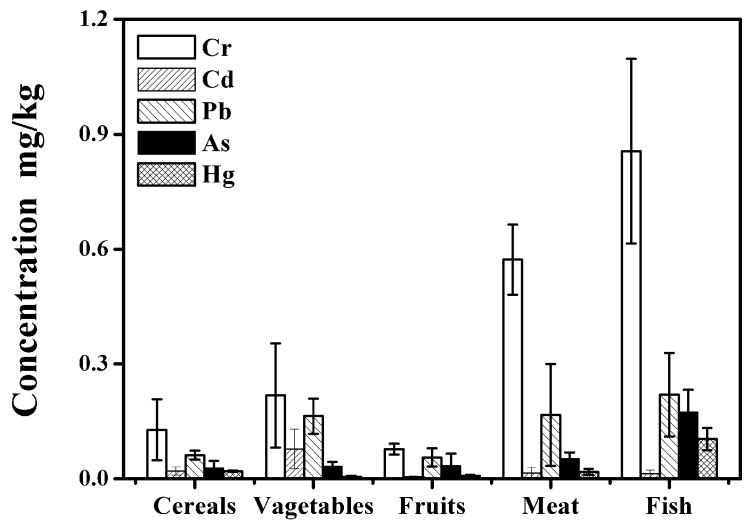
Heavy metal concentrations in different foodstuff species.

The mean metal concentrations in cereals, fruits, vegetables, meat, and fish are presented in Figure 3. These values are all lower than the maximum allowable concentration of consumed foodstuffs in China (GB2762-2005, GB2762-2012). Therefore, there was no evidence that the foodstuffs are a potential source of the studied heavy metals analyzed for the inhabitants living in Beijing.

The estimated dietary intake and total intakes of each metal through food consumption were calculated for the local inhabitants. Dietary daily intakes for the selected heavy metals were calculated by multiplying the respective concentration in each kind of species by the weight of that species group consumed by an average individual. For inhabitants of the studied areas, the daily consumption rates of foodstuff were 366 g/person/day for cereals, 252 g/person/day for vegetables, 69 g/person/day for fruit, 105 g/person/day for meat, and 45 g/person/day for fish, respectively [45].

To evaluate the health risks derived from the dietary intakes of the selected heavy metals(Cr, As, Cd, Hg, and Pb), the weekly intakes (WIs) were calculated and compared to the respective Provisional Tolerable Weekly Intake (PTWIs) established by the FAO/WHO [46]. The estimated WIs through food consumption were 3.4 μg/kg bw/week for Cd, 24.0 μg/kg bw/week for Cr, 11.1 μg/kg bw/week for Pb, 4.0 μg/kg bw/week for As, and 1.8 μg/kg bw/week for Hg, following the decreased order of WIs_Cr_ > WIs_Pb_ > WIs_As_ > WIs_Cd_ > WIs_Hg_. The calculated WIs were all appreciably below the recommended PTWIs (7 μg/kg bw/week for Cd, 1050 μg/kg bw/week for Cr, 25 μg/kg bw/week for Pb, 15 μg/kg bw/week for As, and 5 μg/kg bw/week for Hg). These results indicated that the heavy metal intakes did not pose a health concern for the local inhabitants, which also confirmed the conclusions inferred from the hair analysis results. The highest contribution to total daily intakes of the analyzed heavy metals corresponded to cereals and vegetable. That is because cereals and vegetables were the top two samples of foodstuffs with high ingestion rates. Thus, the ingestion of cereals and vegetables was thought to be the main route by which heavy metals in the environment create hazardous health effects for local inhabitants. However, there was no correlation between trace element intakes and their corresponding levels in hair as well, which was in accordance with previously reported conclusions [1].

## 4. Conclusions

In conclusion, toxic metal concentrations of Cd, Cr, Pb, Hg, and As in hair and foodstuff samples were analyzed in the present study. The results showed that the levels of the concerned metals were all lower than the upper limit of normal values of China, but the exposure levels of heavy metals to the inhabitants living in Beijing should attract greater attention in the future. Correlation and regression analysis of the metal levels in relation to age and gender suggested that age and gender should not be considered as crucial factors for assessing metal concentrations in human hair. No correlation was found between metals intake and the corresponding levels in hair. We can at least conclude that heavy metals levels in hair are associated with age, gender and consumption intakes. Further studies are needed to assess the relationship between heavy metal levels of each age group and intakes of the corresponding heavy metal.

## Figures and Tables

**Table 1:** Heavy metal concentrations in human hair of Beijing inhabitants.

Metals	Mean Values (mg/kg)	Range (mg/kg)	Published Range (mg/kg) [35]
Cd	0.071 ± 0.032	0.02–0.96	0.02–16
Cr	0.782 ± 0.394	0.05–4.54	0.03–33
Pb	1.557 ± 0.779	0.17–6.65	0.004–95
As	0.127 ± 0.078	0.03–0.87	0.015–26
Hg	0.284 ± 0.094	0.04–0.87	0.07–106

**Table 2:** Comparison of hair metal levels in different countries (Mean ± S.D. mg/kg).

Country	Cd	Cr	Pb	As	Hg	Reference
Poland	0.114 ± 0.14	0.568 ± 1.04	1.046 ± 1.39	0.044 ± 0.11	0.500 ± 0.39	[18]
Japan	-	0.31 ± 0.45	4.80 ± 4.64	0.23 ± 0.49	3.18 ± 3.40	[21]
France ^a^	0.011	0.20	0.41	0.05	0.66	[23]
Turkey	-	-	14.11 ± 4.64	-	0.43 ± 0.29	[25]
Sweden	0.058 ± 0.056	0.167 ± 0.118	0.960 ± 0.850	0.085 ± 0.054	0.261 ± 0.145	[35]
Italy	0.23 ± 0.55	0.99 ± 2.17	7.11 ± 5.92	0.09 ± 0.11	-	[36]
Brazil	0.059 ± 0.05	<0.3	12.50 ± 0.70	<0.04	0.620 ± 0.002	[37]
Spanish	0.022 ± 0.054	0.535 ± 0.130	1.46 ± 0.208	0.011 ± 0.007	1.80 ± 1.08	[1]
India ^a^	0.13	-	4.65	2.29	0.82	[38]
Beijing *	0.071 ± 0.032	0.782 ± 0.394	1.557 ± 0.779	0.127 ± 0.078	0.284 ± 0.094	This study

^a^ Median value; * This study.

**Table 3:** Element-to-element correlation coefficients matrix.

Caption	Cd	Cr	Pb	As	Hg
Cd	1				
Cr	−0.024	1			
Pb	0.182 *	0.1	1		
As	0.099	0.156 *	0.399 **	1	
Hg	−0.720	−0.116	−0.147 *	−0.106	1

* Correlation is significant at the 0.05 level (2-tailed); ** Correlation is significant at the 0.01 level (2-tailed).
